# Effects of Dietary Lycopene on the Growth Performance, Antioxidant Capacity, Meat Quality, Intestine Histomorphology, and Cecal Microbiota in Broiler Chickens

**DOI:** 10.3390/ani14020203

**Published:** 2024-01-08

**Authors:** Hongzhi Wu, Sibo Wang, Jiajun Xie, Fengjie Ji, Weiqi Peng, Jinyu Qian, Qian Shen, Guanyu Hou

**Affiliations:** 1 Tropical Crop Genetic Resource Research Institute, Chinese Academy of Tropical Agricultural Sciences, Haikou 571101, China; 2 Abna Management (Shangai) Co., Ltd., Shanghai 200050, China; 3 College of Animal Science and Technology, Northeast Agricultural University, Harbin 150030, China; 4 Hainan Xuhuai Technology Co., Ltd., Haikou 571127, China

**Keywords:** feed additive, dietary lycopene, broiler chickens, growth performance, cecal microbiota

## Abstract

**Simple Summary:**

The Food and Agriculture Organization of the United Nations, the World Health Organization, and the Commission on Food Additives recognize lycopene as a Class A nutrient, but relatively few systematic studies have been conducted on lycopene regulation of antioxidant and meat quality improvement and intestine microbiota in poultry. This study investigated the dietary lycopene from the effects on growth performance, antioxidant capacity, meat quality, intestine histomorphology, and cecal microbiota in broiler chickens. Compared with the control group, the dietary lycopene increased the average daily gain and decreased the feed conversion ratio in the experimental groups; the drip loss_24h_ were decreased in breast muscles in the group treated with 20 mg/kg dietary lycopene; the jejunum villous height was increased in the groups treated with 20 mg/kg dietary lycopene; 13,223 OTUs were identified, each representing a microbial species, and the control group; the Unclassified-f-Ruminococcaceae relative abundance was increased in the groups treated with 20 mg/kg dietary lycopene. In this study, the 20 mg/kg dietary lycopene improved the growth performance, antioxidant capacity, meat quality, intestine histomorphology, and cecal microbiota in broiler chickens. This study provided a theoretical basis for the application of lycopene in poultry production.

**Abstract:**

The experiment aimed to investigate the effects of dietary lycopene on the growth performance, antioxidant capacity, meat quality, intestine histomorphology, and cecal microbiota in broiler chickens. We randomly divided five hundred and seventy-six one-day-old male broilers into four groups each with six replicates and 24 chickens in each replicate. The control group (CG) was fed the basal diet, and the other groups were given powder lycopene of 10, 20, and 30 mg/kg lycopene (LP10, LP20, and LP30, respectively). Compared with the control group, (1) the dietary lycopene increased (*p* = 0.001) the average daily gain and decreased (*p* = 0.033) the feed conversion ratio in the experimental groups; (2) the glutathione peroxidase enzyme contents in LP20 were higher (*p* =< 0.001) in myocardium; (3) the crude protein contents were higher (*p* = 0.007) in the group treated with 30 mg/kg dietary lycopene; (4) the jejunum villous height was higher (*p* = 0.040) in LP20; (5) the Unclassified-f-Ruminococcaceae relative abundance was significantly higher (*p* = 0.043) in LP20. In this study, adding 20 mg/kg dietary lycopene to the broiler chickens’ diets improved the growth performance, antioxidant capacity, meat quality, intestine histomorphology, and cecal microbiota in broiler chickens.

## 1. Introduction

During poultry production, dietary mycotoxin contamination, excessive heavy metals, lipid oxidation, and abuse of antibiotics may lead to poultry intestinal microbiota imbalance. This results in oxidative stress of poultry, causing oxidative damage to the body, reducing their growth performance, meat and egg quality, and ultimately reducing the benefits of poultry breeding [1,2,3]. Lycopene is a red pigment in tomatoes, tomato products, red vegetables, and fruits [4]. It is a polyunsaturated aliphatic hydrocarbon and an acyclic isomer of β-carotene [5]. In addition to its antioxidant and coloring properties, lycopene is classified as a Class A nutrient by the Commission on Food Additives, the Food and Agriculture Organization of the United Nations, and the World Health Organization [6]. Lycopene is the most potent antioxidant among the carotenoids, with the ability to scavenge singlet oxygen 2–3 times that of β-carotene and 100 times that of vitamin E [7]. Lycopene acts as an antioxidant through both physical and chemical means [8]. Its primary physical way is through the direct quenching of singlet oxygen, while its chemical way is through the activation of certain endogenous antioxidant enzymes, such as superoxide dismutase, catalase, glutathione peroxidase, and glutathione reductase; it can also react directly with ROS fragments, such as H_2_O_2_ and NO_2_, to act as a free radical scavenger [9,10]. Lycopene can effectively inhibit the production of hepatocyte pigment P450 in rats with fatty liver model to reduce the lipid peroxidation occurrence [11], and it can also inhibit the production of ROS by regulating the NADPH oxidase activity in mononuclear macrophages [10]. When the antioxidant capacity of a chicken is poor, in that case, its lipids and cholesterol are prone to oxidation, resulting in decreased meat color and tenderness, increased drip loss, odor, and a shortened shelf life [12]. Rozbicka-Wieczorek et al. [13] reported that lycopene improved the antioxidant capacity and tenderness of meat. Dietary plant additives effectively maintain animals’ intestinal microbiota homeostasis, intestinal health, and disease prevention [14]. Tomatoes are widely grown worldwide; therefore, lycopene is widely available. With the banning of antibiotics, lycopene, due to its biological function, not only protects animals at the source but also effectively prevents and inhibits animal diseases, thus reducing the economic losses of farming [15]. At present, the application of lycopene is mainly concentrated in the fields of medicine and food. In contrast, the application of lycopene in animal diets is less studied, and the application status in different animals and feeding stages is unclear.

In this study, we will investigate the effects of lycopene on the growth performance, organ tissue antioxidant capacity, meat quality, intestine histomorphology, and cecal microbiota of arbor acres broiler chickens; explore the feasibility of using lycopene to improve the quality of poultry meat; and provide a theoretical basis for the application of lycopene in poultry production.

## 2. Materials and Methods

### 2.1. Experimental Design and Management

This protocol has been approved by the Northeast Agricultural University’s Animal Care and Use Committee (NEAU-2022-1022). We randomly divided 576 1-day-old male arbor acres broiler chickens into four groups, each with six replicates, and with 24 chickens in each replicate. The control group (CG) was fed the basal diet (corn-soybean diet), and the other groups were given powder lycopene of 10, 20, and 30 mg/kg lycopene (LP10, LP20, and LP30, respectively), which was determined in the preliminary experiment [16]. The lycopene, with a concentration ≥ 96.0%, was bought from Nanjing Jingzhu Biotech. Co., Ltd., Nanjing, China. The basal diet for the broiler chickens was a corn-soybean meal type formulated according to the Agricultural Industry Standard of the People’s Republic of China—Chicken Feed (NY_T33-2004) [17]; the composition of the basal diet and nutritional levels are shown in Table 1 [16]. Corn (granulation), soybean meal (43.00% crude protein content, irregular fragment), corn-protein meal (51.30% crude protein content, microgranular), and cottonseed meal (40.00% crude protein content, irregular fragment) were broken into powder using a grinder (WFJ15, Changzhou Yirui drying equipment Co., Ltd., Changzhou, China), and then mixed with the other ingredients in a blender (JX-100WH, Dongguan Jianxing Environmental Protection Technology Co., Ltd., Dongguan, China). Then, the mixed feed was packed into a double waterproof feed bag for later use. Before the trial, the chicken house was airtightly disinfected using a mixture of formalin and potassium permanganate for three days and ventilated. For 42 days, the birds were reared in battery cages. The humidity of the chicken house was controlled at 60%. The temperature and light time of the chicken house were controlled at 31 °C, 24 h for the first week, and then gradually cooled down to 27 °C, 16 h in the second week, and stabilized at this temperature. The 8-watt light tubes were used to meet the needs of the chicken house for lighting. On the seventh day, the broiler chickens were vaccinated against Newcastle disease using eye drops, and they were inoculated with a bursal polyvalent vaccine using drinking water on the fourteenth day. The broiler chickens were provided with fresh drinking water and were fed twice per day at 8:00 and 17:00. Based on the actual growth of the birds, decide if and when to supplement with a multi-vitamin to their drinking water.

### 2.2. Growth Performance

The body weight of birds was recorded on the first and forty-second day of this experiment to calculate the average daily weight gain (ADG): ADG, g/d = (The body weight on the forty-second day, g—The body weight on the first day, g)/Trial period, d. The average daily feed intake (ADFI) was calculated by recording the daily feeding amount and the feed leftover amount from the previous day: ADFI, g/d = The feed intake in the whole trial period, g/Trial period, d. Moreover, the feed conversion ratio (FCR) was calculated with the ADFI and ADG.

### 2.3. Antioxidant Capacity

On the forty-second day, one broiler chicken with an average body weight in each replicate was selected for slaughter after 12 h of fasting, for collecting the data and samples. Ten grams of the myocardium, breast muscle, and leg muscle in the same part of the broiler chicken were collected and stored at −20 °C to detect the antioxidant capacity parameters, using the HITACHI Automatic Analyzer 3500, Ibaraki-Ken, Japan. The catalog numbers of superoxide dismutase (SOD), total antioxidant capability (T-AOC), glutathione peroxidase enzyme (GSH-Px), and malondialdehyde (MDA) kits were N82750-25g-, GYT0200317, S10152-200UN-, and A003-1, respectively. The reagent kits used in this trial were provided by the Nanjing Jiancheng Biotechnology Co., Ltd., Nanjing, China.

### 2.4. Meat Quality

About 120 g of the breast and leg muscle, respectively, were collected to evaluate the meat quality, including the total moisture, crude protein, crude fat, pH value, drip loss, cooking loss, shear force, and meat color (a*, b*, L*). A portable plug-in pH meter (Testo205, Shanghai, China) was used to measure the breast and leg muscles’ pH value at 45 min and 24 h after slaughter. The a*, b*, and L* of the breast and leg muscles were measured using a portable flesh colorimeter (YD-630, Xiamen, China). The breast and leg muscles were cut into 3 cm × 2 cm × 2 cm, and then the meat was fixed with a fishing line so that the muscle fibers were vertically downward. The samples were hung vertically in the refrigerator at 4 °C for 24 h, and then the juice on the surface of the meat was wiped with filter paper; the weight change was the drip loss [18,19]. After 45 min of heating at 80 °C in a water bath, the breast and leg muscles were cooled at room temperature; the weight change was the cooking loss [20]. Take the muscle thickness of 2.54 cm, water bath heating of the meat to a temperature center of 72–75 °C, natural cooling or a low temperature to reach a specific temperature, along the muscle fiber direction of a diameter of 1.27 cm of the meat column, and then cut off the meat column along the muscle fiber direction using a C-LM3B digital display muscle tenderness instrument (Harbin, China) to get the shear force [21]. The breast and leg muscles were weighed and put into an aluminum box; after roasting in the oven at 120 °C for 15 min, the temperature was adjusted to 65 °C and baked to a constant weight. Then, the samples were crushed through a 40-mesh sieve to make air-dried samples. After extracting the muscle with petroleum ether, the crude fat was obtained by steaming the solvent. The crude fat content was calculated after extraction with petroleum ether using an automatic fat extraction instrument (XD-SXT-210, Shanghai, China). Crude fat in muscle, %, = (Container and fat weight, g − Container weight, g)/muscle weight, g × 100. The crude protein content was determined using an automatic Kjeldahl nitrometer (NKB3100, Shanghai, China). Under the catalyst action, the muscle samples were digested by sulfuric acid, the nitrogen-containing compounds were converted into ammonium sulfate, and alkali distillation was added to allow the ammonia to escape. After absorption by boric acid, the nitrogen content was measured by the standard titrant of hydrochloric acid, and the crude protein content was calculated by multiplying by 6.25. The total moisture content of the fresh meat samples was calculated using the drying method [22]. 

### 2.5. Intestine Histomorphology and Cecal Microbiota

After slaughter, 1 cm jejunum and ileum fragments of the broiler chickens were rinsed with normal saline and fixed with 10% formaldehyde solution for 24 h. Formalin-soaked tissues were embedded in paraffin, cut into 2 mm^2^ sections, stained with hematoxylin-eosin, and photographed at 400× an optical microscope. Motic Images Advanced 3.2 pathological image analysis system (Xiamen, China) was used to read the villus height and crypt depth of the intestinal tract. Each photograph was captured with consistent background lighting. The villus height was measured from the tip to the base (the junction between the villus and crypt), and from the villus junction to the far-end border of the crypt, the crypt depth was measured. Based on the parameters mentioned above, we calculated the villus height/crypt depth (V/C). 

### 2.6. Cecal Microbiota

An amount of 5 g of cecal contents of broiler chickens was collected into a frozen storage tube and stored at −80 °C, and 16s rDNA high-throughput sequencing was performed to analyze the cecal microbial composition in Shanghai Majorbio Bio-pharm Technology Co., Ltd. (Shanghai, China). The data were analyzed using the online platform of the Majorbio Cloud Platform (www.majorbio.com, accessed on 2 July 2023). QIAamp DNA stool mini kits (QIAGEN, CA, Hamburg, Germany) were used to extract total DNA from the cecal contents. The average DNA purity measured as A230/A260 was 2.3, and the 100 μg/mL of average DNA concentration was used for a future test. The 16S rDNA V3–V4 region was amplified by PCR, and the upstream primer 341F (5′-CCTAYGGRBGCASCAG-3′) and the downstream primer 806R (5′-GGACTACNNGGGTATCTAAT-3′) were used for sequence amplification. In this experiment, Phusion@ High-Fidelity PCR Master Mix with GC Buffer (New England Biolabs, Ipswich, MA, USA) enzymes and buffers were used. A 20.00 μL reaction system with 4.00 μL of 5 × FastPfu Buffer, 0.80 μL of forward and reverse primers, 2.00 μL of 2.5 mM dNTPs, 0.40 μL of FastPfu Polymerase, and 10.00 μL of DNA template was used in PCR. During thermal cycling, an initial denaturation at 98 °C for 1 min was followed by 30 cycles of denaturing for 10 s, annealing at 50 °C for 30 s, elongating for 60 s, and finally cooling for 5 min at 72 °C. The 2% agarose gel was used to detect the PCR product purity, and the products were purified using the GeneJET DNA Gel Extraction kit (Thermo Fisher Scientific, Waltham, MA, USA). The library was established using the TruSeq^®^ DNA PCR-Free Sample Preparation Kit, quantified and verified by Qubit, and sequenced using the Illumina Novaseq 6000 platform (Illumina, San Diego, CA, USA). After the original data were obtained using gene sequencing, the acquired raw data were filtered using fastq software (Version 0.19.6), and reads were assembled using FLASH software (Version 1.2.7) to obtain valid data. The sequenced raw data were deposited into the Sequence Read Archive in NCBI with accession number PRJNA1005094.

### 2.7. Statistical Analysis of Data

Statistical analyses were conducted using the SAS 9.4 statistics software (Kerry, NC, USA). Statistical comparisons of different treatments were performed using a one-way ANOVA or Welch ANOVA after the Kolmogorov–Smirnov and variance homogeneity test (Barteet’s test or Levene’s test). Statistical differences among groups were assessed using the Duncan’s multiple range test. The Dunnett’ T3 test was used when the Welch ANOVA was employed. According to the Chao1, Ace, Shannon, Sobs, and Simpson indices, alpha diversity can be estimated. An assessment of Beta diversity was conducted using a weighted UniFrac distance matrix. Data were expressed as mean ± standard error of the mean and *p* < 0.05 as the significant differences.

## 3. Results

### 3.1. Effects of Dietary Lycopene on Growth Performance in Broiler Chickens

As shown in Table 2, dietary lycopene increased (*p* = 0.001) the average daily gain, and decreased (*p* = 0.033) the feed conversion ratio in the experimental groups, but had no significant effect (*p* = 0.147) on the average daily feed intake compared with the CG.

### 3.2. Effects of Dietary Lycopene on Organ Tissue Antioxidant Capacity in Broiler Chickens

The data on antioxidant capacity in organ tissue are shown in Table 3. The GSH-Px content in LP20 and LP30 was higher (*p* =< 0.001) than in the CG, and the T-AOC content in groups treated with dietary lycopene was higher (*p* = 0.031) than in the control group, but there were no significant effects (*p* = 0.415 and *p* = 0.076, respectively) on the SOD and MDA content among all groups in the myocardium.

There were no significant effects (*p* = 0.343, *p* = 0.969, *p* = 0.957, and *p* = 0.834, respectively) on the GSH-Px, SOD, T-AOC, and MDA content among all the groups in the breast muscles. The GSH-Px, SOD, and T-AOC content in the group treated with 30 mg/kg dietary lycopene was higher (*p* = 0.002, *p* =< 0.001, and *p* =< 0.001, respectively) than in the CG, and the MDA content in LP10, LP20, and LP30 was lower (*p* = 0.001) than in the CG in the leg muscles.

### 3.3. Effects of Dietary Lycopene on Muscle Nutrition in Broiler Chickens

**Figure 1:**
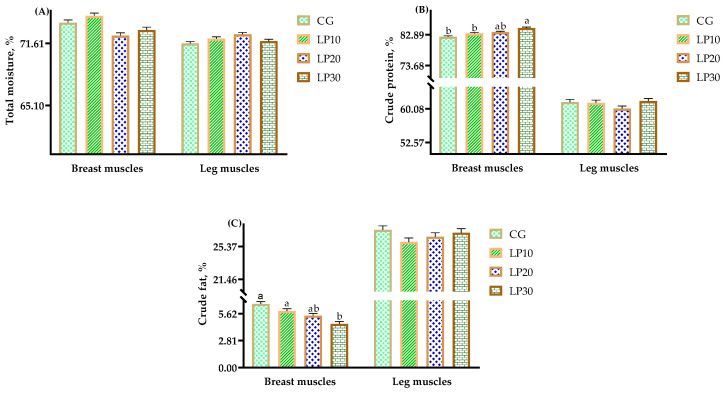
Effects of dietary lycopene on muscle nutrition in broiler chickens. Note: In breast muscles, the data in CG, LP10, LP20, and LP30 were: (**A**) total moisture, %, 73.75, 74.47, 72.40, 72.99, respectively, SEM = 0.30, *p* = 0.078; (**B**) crude protein, %, 82.26^b^, 83.27^b^, 83.63^ab^, 84.87^a^, respectively, SEM = 0.29, *p* = 0.007; (**C**) crude fat, %, 6.64, 5.89, 5.41, 4.56, respectively, SEM = 0.24, *p* = 0.012. In leg muscles, the data in CG, LP10, LP20, and LP30 were: (**A**) total moisture, %, 71.59, 72.09, 72.54, 71.83, respectively, SEM = 0.20, *p* = 0.395; (**B**) crude protein, %, 61.58, 61.42, 60.13, 61.80, respectively, SEM = 0.59, *p* = 0.779; (**C**) crude fat, %, 27.39, 25.90, 26.53, 27.01, respectively, SEM = 0.48, *p* = 0.760. ^a,b^ Means within a row with no common superscripts differ significantly (*p* < 0.05).

The nutrition of breast and leg muscles is shown in Figure 1. The crude protein content was higher (*p* = 0.007), and the crude fat content was lower (*p* = 0.012) in the group treated with 30 mg/kg dietary lycopene than in the CG; there were no significant effects (*p* = 0.078) on the total moisture among all groups in the breast muscles. There were no significant effects (*p* = 0.395, *p* = 0.779, and *p* = 0.760, respectively) on the total moisture, crude protein, and crude fat content among all groups in the leg muscles.

### 3.4. Effects of Dietary Lycopene on Muscle Physical Indicators in Broiler Chickens

The physical indicators of the breast and leg muscles are shown in Table 4. The drip loss_24h_ in LP30 was lower (*p* = 0.030) than in the CG; there were no significant effects (*p* = 0.115, *p* = 0.118, *p* = 0.484 and *p* = 0.432, respectively) on the pH_45min_, pH_24h_, cooking loss, and shear force among all groups in the breast muscles. The shear force in the groups treated with 10 and 20 mg/kg dietary lycopene was lower (*p* = 0.042) than in the CG; there were no significant effects (*p* = 0.292, *p* = 0.463, *p* = 0.878, and *p* = 0.478, respectively) on the pH_45min_, pH_24h_, drip loss_24h_, and cooking loss_24h_ among all groups in the leg muscles.

### 3.5. Effects of Dietary Lycopene on Muscle Color in Broiler Chickens

The muscle color of the breast and leg muscles is shown in Table 5. The breast muscle L* values in LP20 and LP30 were lower (*p* = 0.017) than those in CG; there were no significant effects (*p* = 0.528 and *p* = 0.118, respectively) on the a* and b* values of the breast muscles among all the groups at 24 h after slaughter. There were no significant effects on the L*, a*, and b* values in the breast muscles among all the groups at 45 min (*p* = 0.077, *p* = 0.777, and *p* = 0.518, respectively) after slaughter and in the leg muscles among all the groups at 45 min (*p* = 0.838, *p* = 0.868, and *p* = 0.878, respectively) and 24 h (*p* = 0.162, *p* = 0.423, and *p* = 0.135, respectively) after slaughter. 

### 3.6. Effects of Dietary Lycopene on Intestine Histomorphology in Broiler Chickens

**Figure 2:**
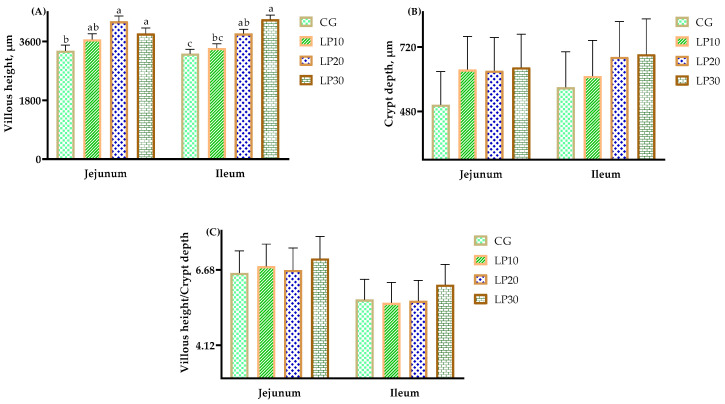
The effects of dietary lycopene on intestine histomorphology in broiler chickens. Note: In jejunum, the data in CG, LP10, LP20, and LP30 were: (**A**) villous height, μm, 3319^b^, 3666^ab^, 4215^a^, 3846^a^, respectively, SEM = 168.08, *p* = 0.040; (**B**) crypt depth, μm, 505, 636, 632, 644, respectively, SEM = 123.61, *p* = 0.204; (**C**) villous height/crypt depth, 6.57, 6.80, 6.67, 7.06, respectively, SEM = 0.75, *p* = 0.747. In ileum, the data in CG, LP10, LP20, and LP30 were: (**A**) villous height, μm, 3230^c^, 3402^bc^, 3848^ab^, 4277^a^, respectively, SEM = 128.04, *p* = 0.003; (**B**) crypt depth, μm, 557, 612, 683, 693, respectively, SEM = 132.34, *p* = 0.489; (**C**) villous height/crypt depth, 5.67, 5.56, 5.63, 6.17, respectively, SEM = 0.69, *p* = 0.118. ^a,b,c^ Means within a row with no common superscripts differ significantly (*p* < 0.05).

The data on intestinal morphology and structure are shown in Figure 2. The jejunum villous height was higher (*p* = 0.040) in the groups treated with 20 and 30 mg/kg dietary lycopene than in the control group; there were no significant effects (*p* = 0.204 and *p* = 0.474, respectively) on the crypt depth and V/C among all the groups in the jejunum. The ileum villous height was higher (*p* = 0.003) in the groups treated with 30 mg/kg dietary lycopene than in the control group; there were no significant effects (*p* = 0.489 and *p* = 0.118, respectively) on the crypt depth and V/C among all the groups in the ileum.

### 3.7. Effects of Dietary Lycopene on Cecal Microbiota in Broiler Chickens

**Figure 3:**
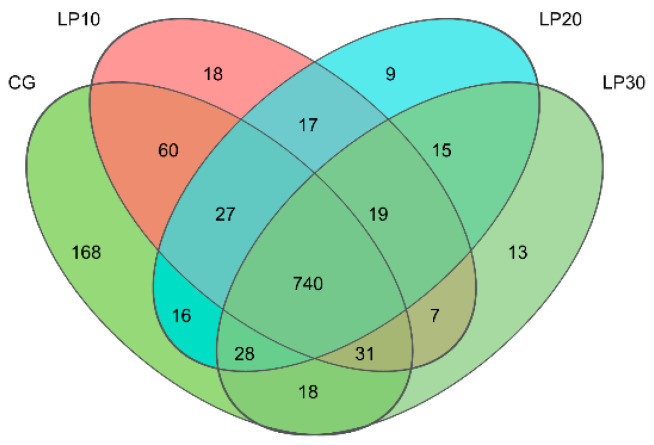
Comparison of operational taxonomic units (OTUs) among groups. CG: Con group; LP10: Group treated with 10 mg/kg lycopene; LP20: Group treated with 20 mg/kg lycopene; LP30: Group treated with 30 mg/kg lycopene.

Cluster analysis was conducted on the clean reads of all samples, and a 97% similarity threshold was used for clustering sequences into operational taxonomic units (OTUs). A total of 13,223 OTUs were identified, each representing a microbial species, and the control group and the treated groups with dietary lycopene shared 740 OTUs. The four CG, LP10, LP20, and LP30 groups were exclusive to 168, 18, 9, and 13 OTUs (Figure 3). The coverage indexes in all groups were more significant than 0.99, indicating that the sequences detected in the samples were comprehensive. The differences in community diversity, Sobs, Shannon, Simpson, Ace, and Chao indices among the groups were insignificant (*p* = 0.691, *p* = 0.418, *p* = 0.559, *p* = 0.617 and *p* = 0.611, respectively) (Table 6). 

**Figure 4:**
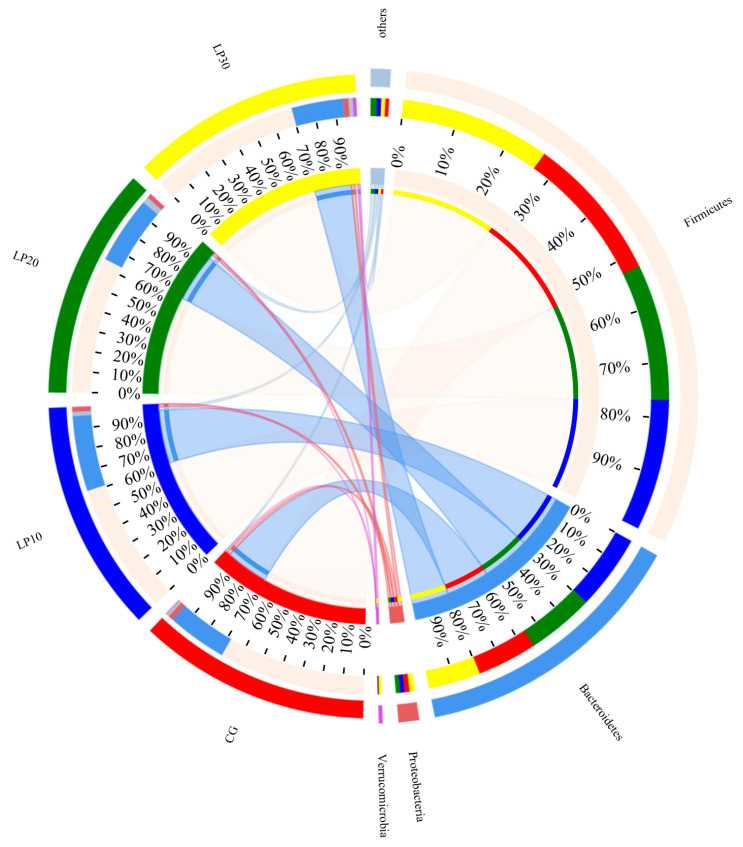
Differences of microbial species in different treatment groups at phylum levels. CG: Con group; LP10: Group treated with 10 mg/kg lycopene; LP20: Group treated with 20 mg/kg lycopene; LP30: Group treated with 30 mg/kg lycopene.

**Figure 5:**
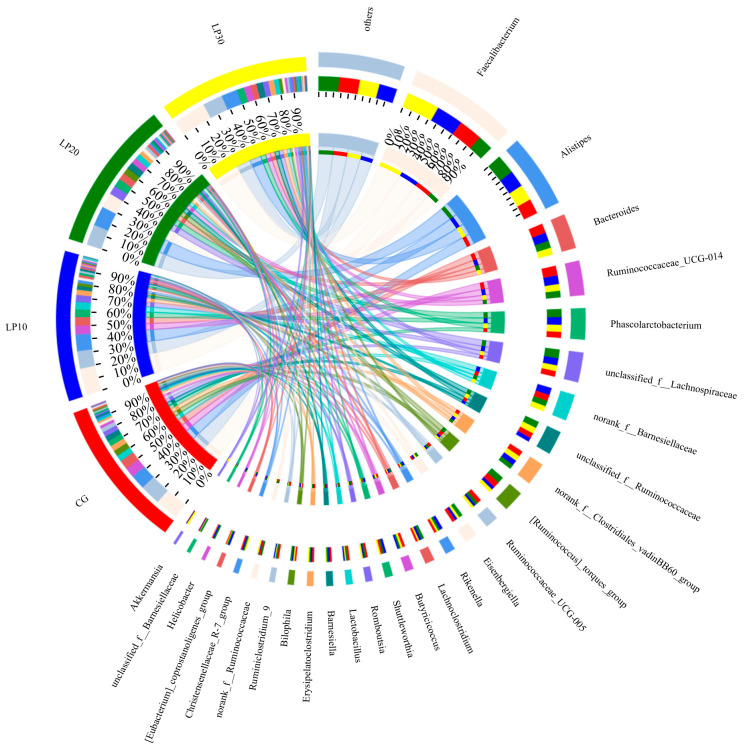
Differences of microbial species in different treatment groups at genus levels. CG: Con group; LP10: Group treated with 10 mg/kg lycopene; LP20: Group treated with 20 mg/kg lycopene; LP30: Group treated with 30 mg/kg lycopene.

The distribution was uniform between the control and treated groups. The microorganisms in the samples mainly consisted of the phylum Firmicutes, Bacteroides, Proteobacteria, Epsilonbacteraeota, Tenericutes, Synergistetes, Verrucomicrobia, and Actinobacteria. The composition of the cecum flora of broiler chickens was dominated by the Firmicutes and Bacteroidets: the Firmicutes’ relative abundance was 69.98%, 67.33%, 64.43%, and 66.45%, respectively (*p* = 0.519); the Bacteroidets’ relative abundance was 24.45%, 28.09%, 25.91%, and 23.83% in the CG, LP10, LP20, and LP30, respectively (*p* = 0.426) (Table 7 and Figure 4). The cecum microorganisms were mainly composed of Faecalibacterium, Alistipes, Ruminococcaceae_UCG, and Unclassified-f-Lachnospiracese after sequence comparison; these four genera in the CG, LP10, LP20, and LP30 accounted for 31.64%, 43.56%, 26.71%, and 34.16% of the total flora, respectively. The Unclassified-f-Ruminococcaceae relative abundance was significantly higher (*p* = 0.043) in the LP20 and LP30 than in the LP10, accounting for 4.02% and 4.55% of the flora, respectively (Table 7 and Figure 5). 

## 4. Discussion

### 4.1. Effects of Dietary Lycopene on the Growth Performance in Broiler Chickens

Plant extracts, such as isoflavones, polyphenols, and carotenoids, each with different molecular structures and functions, could all improve the growth performance of poultry. Riley et al. [23] found that β-carotene improved the final body weights, feed intake, average daily gain, and feed conversion of Ross 308 broiler chickens. Azzam et al. [24] found that isoflavone improved the average daily gain and average daily feed intake of male broiler chickens. Furthermore, Magdalena et al. [25] reported that polyphenols improved the growth performance parameters of broiler chickens, including body weight, body weight gain, and feed intake. Tomato has a large yield as a common vegetable, and its extract, lycopene, has gradually become the focus of attention in recent years. Lycopene could also improve poultry’s growth performance [26]. Fathi et al. [27] found that the diets without lycopene supplementation caused a reduction in feed intake and weight gain and increased the broiler chicken feed conversion ratio with cold-induced ascites. Hosseini-Vashan et al. [28] reported that 5% dried tomato pomace supplementation increased body weight and decreased the feed conversion ratio of heat stress-exposed broiler chickens during 1–28 days of age. In this study, lycopene increased the average daily gain and reduced the feed conversion ratio. It tended to increase the average daily feed intake with increasing lycopene content, which was generally consistent with the above results. This may be related to the fact that lycopene has a highly unsaturated long-chain olefin molecular structure, which can inhibit singlet oxygen, thus improving the antioxidant stability and the growth performance of poultry.

### 4.2. Effects of Dietary Lycopene on the Organ Tissue Antioxidant Capacity in Broiler Chickens

Poultry antioxidant enzyme systems maintain optimal redox homeostasis, which regulates many processes, including cell signaling, gene expression, stress response, and homeostasis [29]. The glutathione peroxidase activities, superoxide dismutase activities, total antioxidant capability, and malondialdehyde content are commonly used indicators to evaluate the antioxidant capacity [30]. Glutathione peroxidase is a peroxidolytic enzyme that catalyzes the conversion of reduced glutathione to oxidized glutathione, protecting the structural and functional membranes of cells from peroxisomal interference and damage [31]. Superoxide dismutase is the main factor in the first level of cellular antioxidant defense [32,33]. $O_{2}^{-}$ is the primary free radical produced in the physiological state of cells. Superoxide dismutase converts $O_{2}^{-}$ to H_2_O_2_ by enzymatic action, and glutathione peroxidase then converts H_2_O_2_ to O_2_ and H_2_O to prevent oxidative damage to the tissues [33]. Total antioxidant capacity refers to the total antioxidant level composed of various antioxidant substances and enzymes, which can be used to evaluate the antioxidant capacity of bioactive substances [34]. Malondialdehyde is a commonly used indicator to measure the degree of oxidative stress [35]. As a lipophilic unsaturated carotenoid, lycopene has a highly unsaturated long-chain olefin molecular structure and the ability to inhibit singlet oxygen, thus rapidly eliminating reactive oxygen species and exerting antioxidant stability [15]. 

Hidayat et al. [36] found that the lycopene in feed significantly decreased the malondialdehyde content and increased the total activities of superoxide dismutase, glutathione peroxidase, total antioxidant capability, and catalase in the meat of broiler chickens. Sun et al. [37] reported that lycopene improved liver total antioxidant capacity, glutathione peroxidase, and glutathione to oxidized glutathione ratio, and significantly decreased liver malondialdehyde levels in chicks. Hosseini-Vashan et al. [28] reported that the activities of glutathione peroxidase and superoxide dismutase were increased, and that the malondialdehyde concentration was decreased in the broiler chickens treated with 5% tomato pomace. Carreras et al. [38] found that the muscle antioxygenic property of broiler chickens fed β-carotene-supplemented diets was significantly enhanced. In this study, the glutathione peroxidase activities, superoxide dismutase activities, and total antioxidant capability contents in LP30 were higher than those in CG, and the malondialdehyde contents in LP10, LP20, and LP30 were lower than those in CG in the leg muscles. This is generally consistent with the results of the above study, which also illustrate that lycopene can improve the antioxidant capacity of broiler chicken organs. 

### 4.3. Effects of Dietary Lycopene on the Meat Quality in Broiler Chckens 

Meat quality is an important economic indicator generally evaluated regarding sensory quality, processing quality, and nutritional level [39]. The pH value indicates the degree of chicken fatty acid, which affects the meat color, tenderness, cooking loss, and nutrient preservation of the chicken after slaughter [40]. The difference in the intramuscular fat content will make the chicken taste and nutritional quality different [41]. Water-holding capacity represents the ability of muscle tissue to retain water, which can directly affect the meat color and tenderness [42]. Shear force can assess the meat tenderness [43]. The breast muscle drop loss_24h_ in the 30 mg/kg dietary lycopene group and the leg muscle shear force was reduced in the 10 mg/kg and 20 mg/kg dietary lycopene groups. Lycopene may affect muscle fiber formation and type [44]. Myofibrillar fiber and connective tissue are the main factors determining meat tenderness. Lycopene can reduce the lactate dehydrogenase and pyruvate kinase activities in the muscle cells and affect the mutual conversion between muscle fiber types [44]. In addition, when muscle protein absorbs more free water, it will expand muscle fibers and loosen connective tissues, which is also conducive to improving meat tenderness [45]. Muscle color depends on myosin oxidation and light reflection from free water [46]. Ma et al. [47] found that dietary lycopene supplementation decreased muscle lightness L* and shear force in broiler chickens. In this study, the breast muscle L* at 24 h after slaughter in the groups treated with 20 mg/kg and 30 mg/kg dietary lycopene was reduced, which may be because, under the action of oxygen, lycopene converted the myoglobin in muscle into oxymyoglobin and continued to be oxidized to ferrimyoglobin, which made the muscle color relatively darker [48]. Muscle proteins adsorb more free water, which also causes them to reflect less light [45]. Lycopene can inhibit ROS attack on cysteine residues in muscle proteins, thereby reducing the rate at which they are converted to disulfide bonds and other thiol oxidation products, ultimately increasing protein synthesis [49]. This may also be why there was increased crude protein content in the group treated with 30 mg/kg dietary lycopene. 3-hydroxy-3-methylglutarate Coenzyme A (HMG-CoA) is a rate-limiting enzyme for lipid biosynthesis, while lycopene can inhibit HMG-CoA synthesis [50]. Napolitano et al. [51] found that lycopene inhibited the synthesis of lipids in single cells. In this study, the breast muscle crude fat content was decreased in the group treated with 30 mg/kg dietary lycopene, which was consistent with the discussion mentioned above.

### 4.4. Effects of Dietary Lycopene on the Intestinal Morphology and Microbiota in Broiler Chickens

The intestinal tract is the most essential animal digestive organ and is crucial in nutrient absorption and digestion [52]. The intestinal structure and function integrity is the prerequisite for nutrient utilization and is also a critical factor in regulating the intestinal flora and establishing the intestinal epithelial immune barrier, as well as one of the indicators to measure its absorption function [53,54]. The cell number in the intestinal epithelium was positively correlated with the villus height. At the same time, the enlarged crypt depth decreased the mature intestinal epithelial cell number, which affected nutrient absorption in the small intestine [55,56]. The results of this experiment showed that the villus height of the jejunum and ileum were increased in the group treated with 20 mg/kg dietary lycopene. This may contribute to the animal growth discussed above, echoing the previous results that lycopene improved the feed conversion ratio and average daily gain in broiler chickens. The reason for the intestinal morphology improvement may be that lycopene stimulates the small intestine to secrete a large number of digestive enzymes and alkaline phosphatase. Alkaline phosphatase is the marker enzyme of the intestinal epithelium, which is positively correlated with the intestinal epithelial differentiation degree and can reflect the degree of intestinal epithelial cell function through its content [57,58]. At the same time, alkaline phosphatase can enhance the absorption of glucose, amino acids, and other nutrients in the intestinal tract of animals, thus promoting animal growth and development [59]. 

The intestinal microbiota controls nutrient absorption, protects against harmful bacteria, and improves growth and metabolism [60]. The intestinal tract is the main organ for microbial colonization; 1-day-old broilers complete microbial colonization after feeding and gradually form a relatively stable microbiota [61]. There are beneficial microorganisms and harmful bacteria in the intestine, of which the unhealthy bacteria number and species are important factors in inducing disease in animals [62]. Under normal circumstances, the microorganisms in the intestine can work together to maintain the internal environment stability and ensure nutrient digestion and absorption in the animal. They can help the animal body to resist the invasion of exogenous pathogens, thus ensuring the animal’s immune system function [60]. In the present study, lycopene was found to have no significant effect on the Sobs, Shannon, Simpson, and Chao1 indices of cecum microorganisms in broilers, suggesting that the dietary lycopene addition had no significant impact on cecum microbial diversity in broiler chickens. Chio et al. [63] found that the dominant phylum of chicken intestinal microbiota was mainly Firmicutes, followed by Bacteroides, Actinobacteria, and Proteobacteria. In this study, Firmicutes, Bacteroides, and Proteobacteria were the dominant phylum in the broiler chickens, suggesting that the microbiota composition of the stable microbiota in the chicken intestine was the same. The Firmicutes phylum makes up the majority of the microbial community in many birds’ intestinal environments [64]. Bacteroides plays a critical role in the decomposition of glucose, lactose, and sucrose, and can also promote the metabolism of nutrients in animals [65]. Turnbaugh et al. [66] showed a correlation between the Firmicutes and Bacteroides ratio (F/B) and lipid metabolism in animals: the smaller the ratio, the lower the fat deposition in the animal. The F/B in the cecum flora in the groups treated with dietary lycopene in this experiment was lower in this study, which may be one of the reasons for the lower muscle crude fat content in the groups treated with dietary lycopene. Ruminococcaceae is one of the most efficient genera of bacteria for breaking down carbohydrates [67]; it plays a crucial role in metabolism with its ability to obtain nutrients by breaking down cellulose in the host’s digestive system [68]. Lachnospiracese is a potentially beneficial bacterium involved in carbohydrate metabolism; it ferments cellulose to produce acetic and butyric acids to provide energy for the host [67,69]. The majority of Epsilonbacteraeota are chemoenergetic autotrophs with diverse metabolic potential and play important roles in the cycling of carbon, nitrogen, and sulfur [70]. In this study, the Unclassified-f-Ruminococcaceae, Unclassified-f-Lachnospiracese, and Epsilonbacteraeota contents in the group treated with 20 mg/kg dietary lycopene were increased, suggesting that lycopene improved the number of beneficial bacteria in the cecum, and echoing the improvement in the average daily gain and feed conversion ratio. 

## 5. Conclusions

In this study, adding 20 mg/kg dietary lycopene to the diet improved growth performance by increasing the average daily gain and decreasing the feed conversion ratio; increased the antioxidant capacity by promoting glutathione peroxidase enzyme and superoxide dismutase levels; promoted the meat quality from the meat color and shear force aspect; ameliorated the intestine health from the histomorphology and cecal microbiota degree in broiler chickens.

## Figures and Tables

**Table 1:** Composition and nutrient content of the basal diet, air-dry basis.

Ingredient	1 to 21 Days	22 to 42 Days
Corn, %	57.85	62.30
Soybean meal (43.00%), %	29.00	24.00
Corn protein meal (51.30%), %	4.60	5.00
Cottonseed meal (40.00%), %	2.00	2.50
Soybean oil, %	2.70	2.80
Dicalcium phosphate, %	1.90	1.65
Limestone, %	1.04	1.00
Sodium chloride, %	0.30	0.30
*L*-Lysine, %	0.09	0.03
Methionine, %	0.20	0.10
Choline chloride, %	0.10	0.10
Multi-vitamin ^1^, %	0.02	0.02
Multi-microelement ^2^, %	0.20	0.20
Total	100.00	100.00
Nutrient levels		
Metabolizable energy ^3^, MJ/kg	12.53	12.76
Crude protein ^4^, %	21.47	19.98
Lysine ^4^, %	1.16	1.00
Methionine ^4^, %	0.53	0.42
Methionine+Cysteine ^4^, %	0.88	0.76
Threonine ^4^, %	0.78	0.73
Tryptophan ^4^, %	0.24	0.21
Calcium ^4^, %	1.01	0.93
Available Phosphorus ^4^, %	0.45	0.37

Note: ^1^ The multi-vitamin provided the following per kg of diets: VA 8000 IU; VD 4000 IU; VE 12 mg; VK_3_ 1.6 mg; VB_1_ 2 mg; VB_2_ 6 mg; VB_6_ 3 mg; VB_12_ 0.012 mg; Nicotinic acid 20 mg; Folic acid 0.8 mg; Biotin 0.04 mg; Pantothenic acid 9 mg. ^2^ The multi-mineral provided the following per kg of diets: Fe as ferrous sulfate 80 mg; Mn as manganese sulfate 100 mg; Zn as zinc sulfate 75 mg; Cu as copper sulfate 8 mg; I as potassium iodide 0.35 mg; Se as sodium selenite 0.15 mg. ^3^ Calculated value. ^4^ Analyzed content.

**Table 2:** Effect of dietary lycopene on growth performance of broiler chickens.

Items	CG	LP10	LP20	LP30	SEM	*p*-Value
ADG, g	51.25 ^b^	58.16 ^a^	59.12 ^a^	58.11 ^a^	0.87	0.001
ADFI, g	92.22	93.78	94.18	93.44	0.41	0.147
FCR	1.79 ^a^	1.61 ^b^	1.59 ^b^	1.61 ^b^	0.19	0.033

Note: ^a,b^ Means within a row with no common superscripts differ significantly (*p* < 0.05). ADG: Average daily gain; ADFI: Average daily feed intake; FCR: Feed conversion ratio.

**Table 3:** Effect of dietary lycopene on organ tissue antioxidant capacity in broiler chickens.

Items	CG	LP10	LP20	LP30	SEM	*p*-Value
Myocardium						
GSH-Px (U/mg prot)	16.93 ^c^	16.47 ^c^	20.03 ^b^	24.18 ^a^	0.75	<0.001
SOD (U/mg prot)	506	439	489	471	14.43	0.415
T-AOC (U/mg prot)	72.64 ^b^	96.01 ^a^	94.56 ^a^	102 ^a^	3.90	0.031
MDA (nmol/mg prot)	0.59	0.47	0.37	0.49	0.03	0.076
Breast muscles						
GSH-Px (U/mg prot)	3.86	2.94	3.81	4.15	0.25	0.343
SOD (U/mg prot)	72.79	75.57	77.36	74.64	3.11	0.969
T-AOC (U/mg prot)	134	133	137	137	3.43	0.957
MDA (nmol/mg prot)	0.14	0.12	0.12	0.13	0.01	0.834
Leg muscles						
GSH-Px (U/mg prot)	21.47 ^b^	19.36 ^b^	22.74 ^b^	33.62 ^a^	1.39	0.002
SOD (U/mg prot)	69.77 ^b^	68.10 ^b^	73.27 ^b^	86.59 ^a^	1.96	<0.001
T-AOC (U/mg prot)	99.06 ^b^	96.11 ^b^	102 ^b^	133 ^a^	3.92	<0.001
MDA (nmol/mg prot)	0.86 ^a^	0.36 ^b^	0.33 ^b^	0.25 ^b^	0.07	0.001

Note: ^a,b,c^ Means within a row with no common superscripts differ significantly (*p* < 0.05). GSH-Px: Glutathione peroxidase enzyme; SOD: Superoxide dismutase; T-AOC: Total antioxidant capability; MDA: Malondialdehyde.

**Table 4:** Effects of dietary lycopene on breast and leg muscle physical indicators in broiler chickens.

Items	CG	LP10	LP20	LP30	SEM	*p*-Value
Breast muscles						
pH_45min_	5.61	5.75	5.67	5.84	0.36	0.115
pH_24h_	5.35	5.34	5.59	5.39	0.42	0.118
Drip loss_24h_, %	3.21 ^a^	2.23 ^ab^	2.55 ^ab^	1.63 ^b^	0.20	0.030
Cooking loss, %	27.18	23.10	23.64	25.96	1.04	0.484
Shear force, kgf	3.82	2.96	3.13	3.42	0.19	0.432
Leg muscles						
pH_45min_	5.50	5.50	5.65	5.60	0.33	0.292
pH_24h_	5.68	5.98	5.83	5.88	0.67	0.463
Drip loss_24h_, %	3.49	3.40	4.03	3.84	0.29	0.878
Cooking loss, %	29.93	26.94	30.32	27.88	0.87	0.478
Shear force, kgf	3.41 ^a^	2.59 ^b^	2.79 ^b^	2.93 ^ab^	0.11	0.042

Note: ^a,b^ Means within a row with no common superscripts differ significantly (*p* < 0.05).

**Table 5:** Effects of dietary lycopene on muscle color in broiler chickens.

Items	CG	LP10	LP20	LP30	SEM	*p*-Value
Breast muscles, 45 min after slaughter						
L*	47.61	51.98	49.71	49.37	0.61	0.077
a*	2.05	2.42	2.37	1.54	0.32	0.777
b*	8.71	8.86	9.53	7.85	0.39	0.518
Breast muscles, 24 h after slaughter						
L*	57.36 ^a^	54.50 ^ab^	51.57 ^b^	52.07 ^b^	0.77	0.017
a*	1.67	1.58	2.23	1.57	0.18	0.528
b*	10.86	10.1	10.66	7.54	0.55	0.118
Leg muscles, 45 min after slaughter						
L*	51.86	53.8	52.6	52.4	0.74	0.838
a*	4.32	3.74	3.83	3.63	0.29	0.868
b*	14.01	13.16	13.99	12.88	0.58	0.878
Leg muscles, 24 h after slaughter						
L*	55.57	53.42	51.89	51.54	0.70	0.162
a*	3.21	4.8	4.92	3.52	0.44	0.423
b*	12.66	9.29	10.62	8.97	0.62	0.135

Note: ^a,b^ Means within a row with no common superscripts differ significantly (*p* < 0.05).

**Table 6:** Effects of dietary lycopene on cecal microbiota alpha diversity in broiler chickens.

Items	CG	LP10	LP20	LP30	SEM	*p*-Value
Sobs	596	575	573	582	59.11	0.691
Shannon	4.41	4.23	4.41	4.39	0.35	0.418
Simpson	0.03	0.05	0.03	0.02	0.02	0.559
Ace	675	656	649	640	69.04	0.617
Chao1	684	671	651	763	69.17	0.611
Coverage	0.998	0.998	0.998	0.998	0.00	0.268

Note: The Sobs item represents the number of species that were actually observed. Using Shannon’s index, a sample’s microbial diversity is estimated on the basis of its richness and evenness. An indicator of community diversity, the Simpson Index calculates the probability that two randomly sampled sequences belong to different species within a community. Ace is an index that measures the degree of species richness and evenness in a sample. The Chao1 Index estimates the number of operational taxonomic units (OTUs) contained in a sample.

**Table 7:** The cecal microbial abundance at the phylum and genus levels in broiler chickens.

Items	CG	LP10	LP20	LP30	SEM	*p*-Value
Phylum level, %						
Firmicutes	69.98	67.33	64.43	66.45	1.13	0.519
Bacteroidets	24.45	28.09	25.91	23.83	1.02	0.426
Proteobacteria	2.42	1.43	1.68	2.68	0.25	0.237
Epsilonbacteraeota	0.68	1.25	3.08	0.75	1.35	0.106
Tenericutes	0.64	0.84	0.70	0.63	0.12	0.076
Synergistetes	0.61	0.47	0.45	0.57	0.20	0.934
Verrucomicrobia	0.27	0.15	2.66	3.57	0.63	0.984
Actinobacteria	0.66	0.29	0.24	0.40	0.23	0.226
Genus level, %						
Faecalibacterium	10.39	18.67	5.25	13.03	2.34	0.246
Alistipes	7.54	10.72	10.91	10.9	0.71	0.271
Ruminococcaceae_UCG	9.55	7.81	3.44	5.78	1.23	0.369
Unclassified-f-Lachnospiracese	4.16	6.36	7.11	4.45	0.49	0.057
Phascolarctobacterium	2.65	6.22	4.22	6.6	0.71	0.161
Bacteroides	5.46	5.11	4.29	3.92	0.52	0.758
Norank-f-Barnesiellaceae	5.26	3.27	2.52	3.72	0.58	0.442
Unclassified-f-Ruminococcaceae	3.55 ^ab^	2.02 ^b^	4.02 ^a^	4.55 ^a^	0.36	0.043
Norank-f-clostridiales	1.77	2.45	2.64	2.68	0.35	0.833

Note: ^a,b^ Means within a row with no common superscripts differ significantly (*p* < 0.05).

## Data Availability

Raw reads of bacterial 16S rDNA gene sequencing are available in the NCBI Sequence Read Archive database (Accession Number: PRJNA1005094). Other data that support the findings of this study were not deposited in an official repository, but they are available from the authors upon request.
